# Morphological and Metabolite Responses of Potatoes under Various Phosphorus Levels and Their Amelioration by Plant Growth-Promoting Rhizobacteria

**DOI:** 10.3390/ijms22105162

**Published:** 2021-05-13

**Authors:** Leangsrun Chea, Birgit Pfeiffer, Dominik Schneider, Rolf Daniel, Elke Pawelzik, Marcel Naumann

**Affiliations:** 1 Department of Crop Sciences, Division Quality of Plant Products, University of Göttingen, Carl-Sprengel-Weg 1, 37075 Göttingen, Germany; leangsrun.chea@agr.uni-goettingen.de (L.C.); epawelz@gwdg.de (E.P.); 2 Department of Genomic and Applied Microbiology, Institute of Microbiology and Genetics, University of Göttingen, Grisebachstraße 8, 37077 Göttingen, Germany; bpfeiff@gwdg.de (B.P.); dschnei1@gwdg.de (D.S.); rdaniel@gwdg.de (R.D.)

**Keywords:** plant biomass, metabolite profiling, mineral nutrients, phosphorus deficiency, phosphorus toxicity, potato, plant growth-promoting rhizobacteria, secondary metabolites

## Abstract

Low phosphorus (P) availability is a major limiting factor for potatoes. P fertilizer is applied to enhance P availability; however, it may become toxic when plants accumulate at high concentrations. Therefore, it is necessary to gain more knowledge of the morphological and biochemical processes associated with P deficiency and toxicity for potatoes, as well as to explore an alternative approach to ameliorate the P deficiency condition. A comprehensive study was conducted (I) to assess plant morphology, mineral allocation, and metabolites of potatoes in response to P deficiency and toxicity; and (II) to evaluate the potency of plant growth-promoting rhizobacteria (PGPR) in improving plant biomass, P uptake, and metabolites at low P levels. The results revealed a reduction in plant height and biomass by 60–80% under P deficiency compared to P optimum. P deficiency and toxicity conditions also altered the mineral concentration and allocation in plants due to nutrient imbalance. The stress induced by both P deficiency and toxicity was evident from an accumulation of proline and total free amino acids in young leaves and roots. Furthermore, root metabolite profiling revealed that P deficiency reduced sugars by 50–80% and organic acids by 20–90%, but increased amino acids by 1.5–14.8 times. However, the effect of P toxicity on metabolic changes in roots was less pronounced. Under P deficiency, PGPR significantly improved the root and shoot biomass, total root length, and root surface area by 32–45%. This finding suggests the potency of PGPR inoculation to increase potato plant tolerance under P deficiency.

## 1. Introduction

Phosphorus (P) is an essential element for plant growth and metabolism [1]. Although P in natural soils is abundant, its availability for plants is very limited [2]. Since the 1950s, inorganic P fertilizer use has rapidly increased to enhance soil P availability in order to increase crop yields [3]. Excessive P application often results in low P use efficiency because the majority of applied P becomes insoluble complexes and is lost through surface run-off, causing eutrophication [2]. Meanwhile, almost 30% of global arable land is P-deficient due to less accessibility to P fertilizers in the regions [3]. Therefore, it is necessary to optimize P fertilization practices to improve P efficiency.

Compared to other crops, potato has a relatively high P demand. If all other nutrients are available in sufficient amount, potato needs a soil P concentration of about 76 mg kg^−1^ of Olsen P, which is four times higher than the demand of cereals to achieve 95% of its yield potential [4]. This high requirement is caused by the shallow root system and inefficiency in P uptake at low soil P concentration [5], making P a major limiting factor in potato production. P is important for early root and shoot development of the plant, and it also contributes most to increasing tuber yield [6]. Continuous supply of inorganic P may result in high P accumulation in the soil, and it subsequently creates P toxicity causing plant biomass reduction [7]. However, assessing responses of potato to low and high P applications is difficult in field or pot conditions. In soils effects of P application can be inhibited by either soil organic P mobilization to balance available P concentration, or complexation of P and metallic ions that reduce availability of applied P [2]. The hydroponic system is a more suitable tool to assess the plant growth and metabolite responses under P deficiency and toxicity [8]. Even if the hydroponic condition does not always reflect the growth conditions of plants in the field, it is widely used to assess the mechanisms underlying plant responses under varying P applications. This is because the interference by organic P solubility and plant–plant interaction, which often occurs in the fields, is omitted or minimized [8,9].

To ensure growth, plants require adaptation mechanisms from plant to cell level to cope with P deficiency and toxicity conditions. Root growth is improved under P deficiency compared to shoot growth, in order to enable the root system to exploit limited available P [2,10]. Deficient and excess P applications also induce nutrient imbalance, which affects the uptake of other nutrients by the plant and influences crop nutritional status [11]. Apart from these morphological and nutritional interferences, P deficiency also regulates plant secondary metabolites such as accumulation of leaf anthocyanin, flavonoids, and amino acids. Stewart et al. [12] reported that flavonoid concentration of Arabidopsis and tomato was four and two times higher when P availability was reduced from 6.3 mM to 0 mM, respectively. Plant stress induced by either P deficiency or toxicity leads also to the accumulation of proline, which helps prevent the plant cellular structures from oxidative damage and triggers the accumulation of the abovementioned secondary metabolites [13,14]. Moreover, modifications of plant metabolic pathways can occur under P deficiency to enhance internal P use [2]. Nguyen et al. [15] found a significant increase in organic acids, but a decrease in sugars and phosphorylated sugars in roots of wheat cultivars under low P supply. However, there is a lack of research regarding the biochemical adaptation of potato under P deficiency. There is also no information available on the P toxicity of potato that elucidates its physiological and metabolic responses, except some reports showing biomass reduction and micronutrient (i.e., iron [Fe], zinc [Zn], and manganese [Mn]) deficiency under excess P conditions [7,11]. Therefore, understanding and characterizing potato response to P deficiency and toxicity is necessary to improve the plant’s tolerance to these conditions.

Furthermore, there has been a growing interest in exploring the potential of plant growth-promoting rhizobacteria (PGPR) in increasing P efficiency. Many PGPR can produce secondary metabolites and phytohormones that can stimulate the hormonal pathways of plants involved in root development [16]. These positive effects of PGPR have been reported in pea [17] and other vegetable crops, including potato [18,19]. Menéndez and Paço [20] demonstrated the benefit of using a diverse set of PGPR instead of single-strain inoculant on plants; however, it requires a deeper look at plant-PGPR interaction. To the best of our knowledge, little studies have been conducted to understand the interactive effect of PGPR and P on root growth and P uptake of potato.

In the present study, a range of P fertilization levels (0–40 mg L^−1^) were tested on potato plants in a hydroponic condition. Five bacterial strains including *Variovorax paradoxus* DSM 30,034 (NBRC 15149), *Azoarcus sp.* DSM 9506, *Azospirillum sp.* DSM 1842, *Bacillus subtilis* DSM 21393, and *Pseudomonas putida* DSM 6125 (KT2440) were co-inoculated mainly at low P levels (0–2 mg L^−1^). These strains were selected based on literature research of their growth-promoting effects on various crop plants including vegetables, field crops, and paddy rice in which the submerged conditions of the latest crop are similar to the hydroponic condition. The objectives of the present study were (I) to assess plant morphology, mineral allocation, and metabolite compounds of potato in response to different P applications and (II) to evaluate how PGPR affects plant biomass, P uptake, and metabolites at low P levels in nutrient solution. Consequently, the present study shows the first on metabolite profiling of potato root under P deficiency and toxicity conditions. The potential application of PGPR for potato root growth enhancement is also elucidated.

## 2. Results

### 2.1. Effect of P Applications on Plant Morphology and Leaf Chlorophyll Concentration

**Figure 1:**
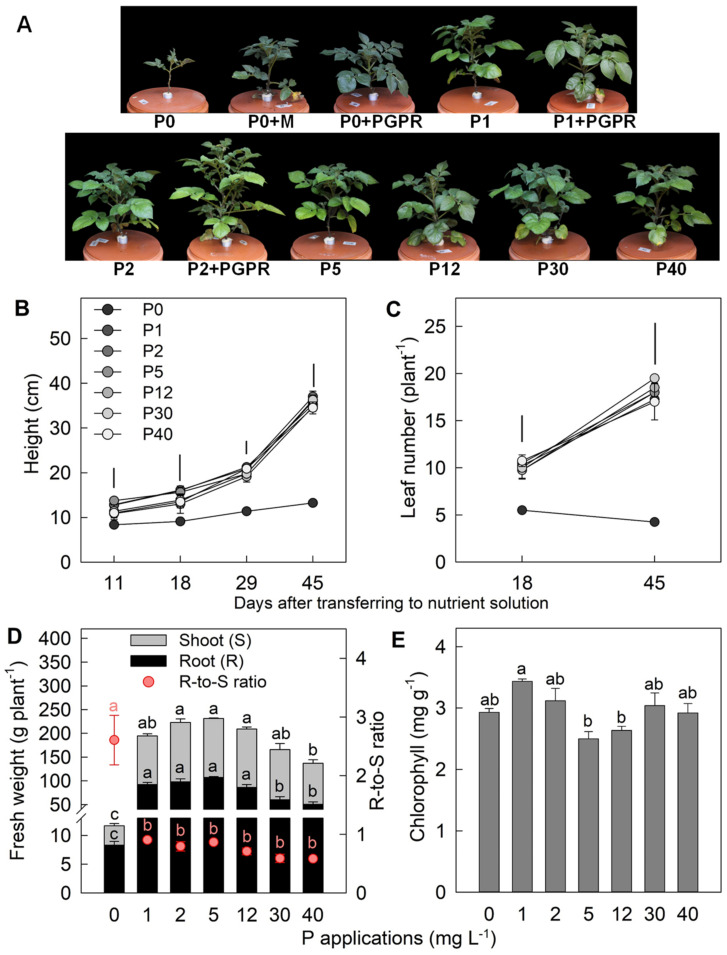
(**A**) Plant shoot phenotype at 30 days after seedling transplanting (DAT) as affected by different P levels and PGPR inoculation and (**B**–**E**) the influence of different P applications (0–40 mg L^−1^) on morphology and leaf chlorophyll concentration of potato plants. Error bar represents standard error of means. Vertical bars in (**B**,**C**) represent critical value for comparisons of plant height and leaf number among P treatments in each measurement date by Tukey’s HSD test at *p* ≤ 0.05. Different letters in (**D**,**E**) of the same parameter indicate significant difference by Tukey’s HSD test at *p* ≤ 0.05.

The important plant morphological traits and leaf chlorophyll were assessed in response to varying P applications in nutrient solution. Plants under P0 (0 mg P L^−1^) showed deficiency symptoms through stagnated height development and significant reduction in leaf number during the growing period (Figure 1A–C). Regarding increasing P applications, root and shoot biomass significantly increased up to P5 (5 mg P L^−1^). Root and shoot biomass of P5 plants were 12- and 36-fold higher, respectively, than that of P0, but they were not significantly different from P1 (1 mg P L^−1^) and P2 (2 mg P L^−1^). Increasing P applications higher than P5 resulted in a significant decrease in root biomass by 44–53%, as well as in shoot biomass by 14–30%, under both P30 (30 mg P L^−1^) and P40 (40 mg P L^−1^) compared to P5 (Figure 1D). Therefore, shoot growth was severely impaired under P limitation (P0). Excess P applications (P30 and P40) strongly inhibited root biomass, implying P toxicity conditions for plants. These results also suggest that P5 may be the optimum P application for potato plant growth in this study. However, leaf chlorophyll concentration increased by 17–20% under both P limitation and excess compared to P5, even though it was not significant (Figure 1E).

### 2.2. Effect of P Applications on Nutrient Concentration in Different Plant Parts

**Figure 2:**
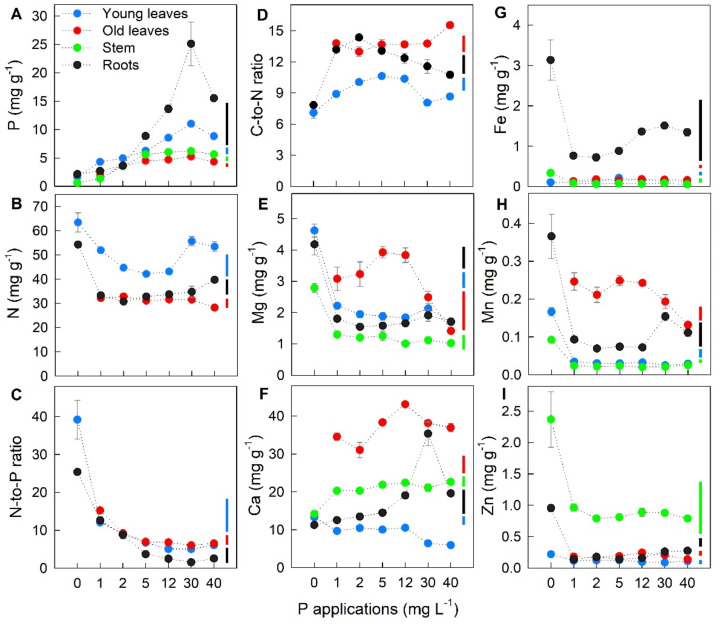
Effect of different P applications on (**A**–**B**,**E**–**I**) mineral concentration and (**C**,**D**) mineral ratios in young leaves, old leaves, stem, and roots. For all measured traits, P0 data for old leaves are missing due to insufficient sample material for analyses. Error bars indicate standard error of means (*n* = 4). Vertical bars represent critical value for comparisons among P applications in each plant part by Tukey’s HSD test at *p* ≤ 0.05.

Young leaves, old leaves, stem, and roots of potato were harvested 45 days after the onset of P treatments for nutrient concentration quantification. Tissue P analyses revealed that P concentration in all plant parts increased at different magnitudes in response to increasing P applications in the nutrient solution up to P30 and, subsequently, decreased at P40 by 20%, 17%, 9%, and 46% in young leaves, old leaves, stem, and roots, respectively, compared to those at P30 (Figure 2A).

Furthermore, Figure 2B–I and Figure S1 show that varying P applications to the nutrient solution also significantly induced different concentrations and distribution patterns of macro- and micronutrients among plant parts. Plants under P0 exhibited 23–82% higher in nitrogen (N), N-to-P ratio, magnesium (Mg), Fe, Mn, and Zn concentration, but 33–60% lower in the carbon-to-nitrogen (C-to-N) ratio in all studied parts compared to plants under optimum P (P5). In response to increasing P applications, Mg, calcium (Ca), Fe, and Mn concentrations in young leaves, old leaves, and stem decreased at P40 by 40–63% compared to those at P5 (Figure 2E–H).

### 2.3. Effect of P Applications on Plant Secondary Metabolites and Amino Acids

**Figure 3:**
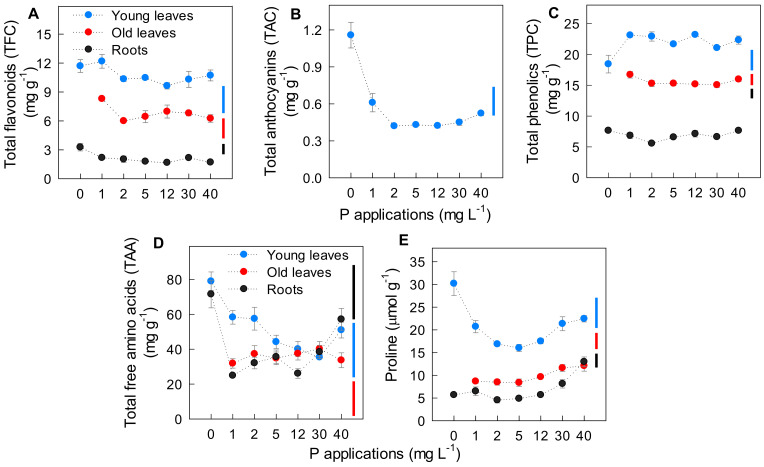
Effect of different P applications on the concentration of (**A**–**C**) secondary metabolites, (**D**) total free amino acids, and (**E**) proline in young leaves, old leaves, and roots. For all measured traits, P0 data of old leaves are missing due to insufficient sample material for analyses. Error bars indicate standard error of means (*n* = 4). Vertical bars represent critical value for comparisons among P applications in each plant part by Tukey’s HSD test at *p* ≤ 0.05.

To confirm P deficiency and toxicity conditions for the potato plant, secondary metabolites and amino acids such as total phenolics (TPC), total flavonoids (TFC), total free amino acids (TAA), and proline concentration in young leaves, old leaves, and roots, and total anthocyanins (TAC) in young leaves were quantified. Figure 3 shows that TFC, TAC, TAA, and proline concentrations in young leaves and roots of plants at P0 were 60–200% higher than in those under P5. However, TAC in young leaves decreased in response to increasing P applications until P5, followed by a subsequent increase at P40 by 25% compared to those at P5. Increasing P applications to the nutrient solution also led to decreasing of TAA and proline in all plant parts, but they recovered in young leaves and roots at P30 with a subsequent increase at P40. This recovery was more evident in roots, in which the TAA and proline concentrations increased by 1.5 and 2 times at P30 and P40, respectively, compared to P5. The high accumulation of these secondary metabolites, especially TAA and proline, at P0, P30, and P40 indicate P deficiency- and toxicity-induced stress conditions in plants. There was no clear indication for P deficiency at P1 and P2, and P toxicity at P12 (12 mg P L^−1^).

### 2.4. Root Metabolite Profiling under P Deficiency and Toxicity Conditions

Since there was a relatively high accumulation of total free amino acids at P deficiency (P0) and P toxicity (P30 and P40) compared with P optimum (P5), a further investigation was conducted on the metabolic changes in root tissue under P-deficient and P toxic conditions in comparison with P optimum using nuclear magnetic resonance (NMR) spectroscopy. A total of 100 metabolites were detected in root tissue, of which 37 were above the detection limit of measurements. These metabolites include amino acids, sugars, organic acids, and other organic compounds (Table S1).

**Figure 4:**
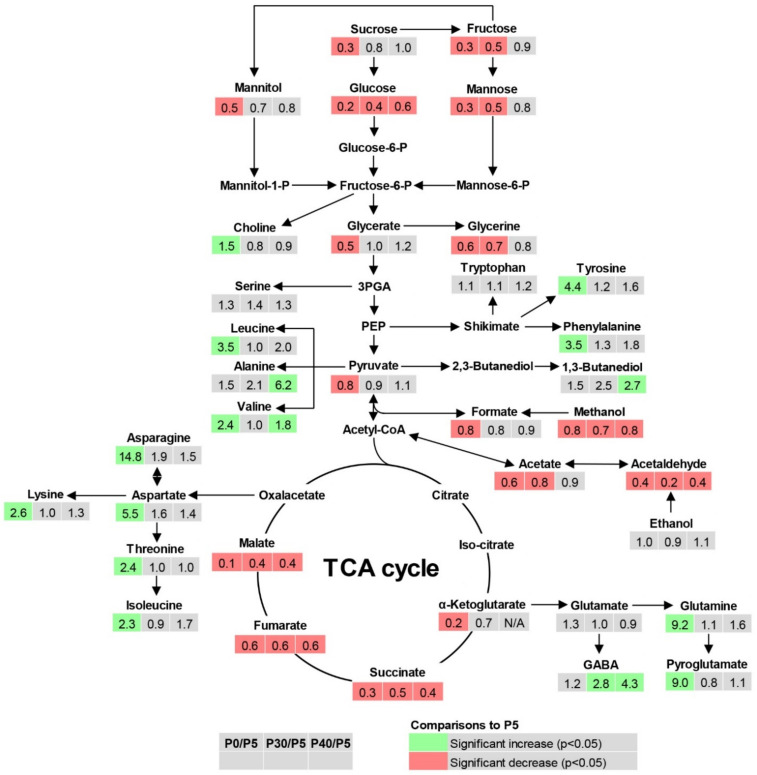
Effect of P deficiency and toxicity on metabolite concentration in roots of potato plants. The relative ratios were calculated by the division of metabolite concentration plant grown at P0, P30, and P40 with those at P5 as P sufficient treatment (*n* = 4). Light green and red present the significant increase and decrease (*p* ≤ 0.05), respectively. N/A = comparison not possible due to incomplete data; 3PGA = 3-phosphoglycerate; PEP = Phosphoenolpyruvate; Acetyl-CoA = Acetyl Coenzyme A; TCA = tricarboxylic acid; GABA = γ-aminobutyrate. Arrows with one direction show synthesis of a metabolite and arrows with double direction show reversible reactions.

Figure 4 shows the pathway and fold changes of metabolites in root tissue under P deficiency and toxicity compared with the optimal P condition. P deficiency significantly reduced sugars including sucrose, fructose, mannitol, and mannose from 50% to 80%. Organic acids generally decreased under P deficiency by 20–90%. These acids included glycerate, pyruvate, formate, acetate, α-ketoglutarate, succinate, fumarate, and malate. In contrast, amino acids significantly increased by 1.5–14.8 times under P deficiency. Of these amino acids, asparagine presented the highest fold change (14.8).

The effects of P toxicity were more evident in organic acids and their precursors than in sugars and amino acids (Figure 4). Toxic P conditions, either at P30 or P40, significantly decreased glycerine, methanol, acetate, acetaldehyde, succinate, fumarate, and malate by 20–80%. The significant decrease in sugars (by 50–60%) under P toxicity was found only for fructose, glucose, and mannose. Similar to P deficiency, P toxicity condition also caused a 1.8–4.3 time increase in amino acids such as alanine, valine, and γ-Amino-butyrate (GABA). 1,3-Butanediol also increased 2.5–2.7 times under P toxicity. Therefore, the conditions of both P deficiency or P toxicity significantly reduced sugar and organic acid levels in the root, but increased amino acids concentration.

### 2.5. Effect of PGPR Inoculation on Plant Growth and Secondary Metabolites at Low P Supplies

**Figure 5:**
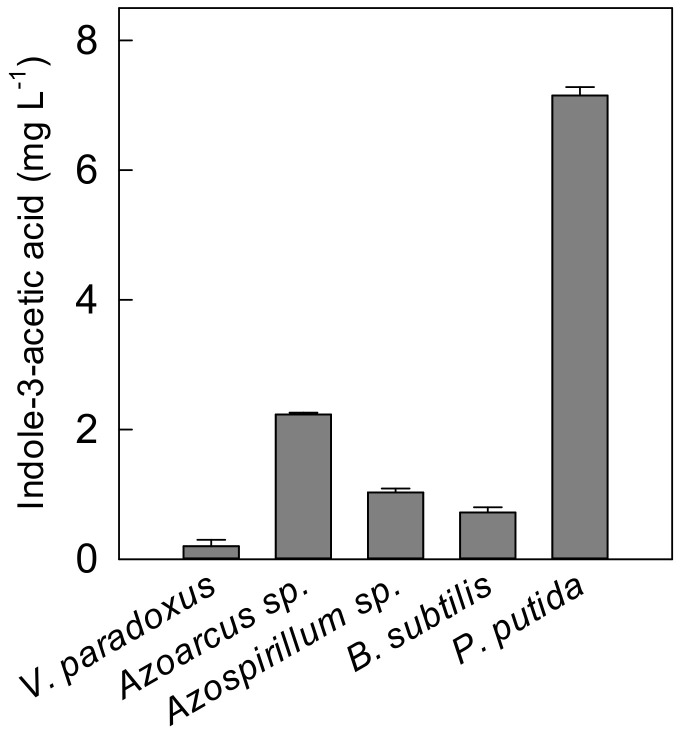
Indole-3-acetic acid (IAA) concentration produced by each bacteria strain in its respectively culture at OD_600_ = 0.4.

To test the hypothesis that PGPR inoculation improves plant growth under low P supply, a mixture of five potential plant growth-promoting strains was co-inoculated in the nutrient solution at P0, P1, and P2. Plants with and without PGPR inoculation were compared to reveal the effect of PGPR under specific P treatment. In addition, a control with added sterile media was conducted under P0. The inoculants and media were renewed once a week. At 1 and 7 days after inoculation, nutrient solution was plated on strain-specific nutrient agar. After a 24-h incubation of plates in room temperature, there was growth of bacteria in which the colonies matched in appearance to the original strain. This observation indicates the viability of the inoculants in nutrient solution at both 1 and 7 days after inoculation. Furthermore, in-vitro determination of indole-3-acetic acid (IAA) indicated that each bacterial strain was able to produce IAA at different levels ranging from 0.2 mg L^−1^ (*Variovorax paradoxus* DSM 30034) to 7.15 mg L^−1^ (*Pseudomonas putida* DSM 6125) (Figure 5). Table 1 shows that the effect of PGPR was observed only on plants under P0 for plant height, leaf number, root biomass, shoot biomass, and total biomass. By considering the media effect at P0, the results revealed that PGPR significantly increased root, shoot, and total biomass by 45%, 33%, and 42%, respectively. Total root length and root surface area of PGPR-inoculated plants were also 44% and 32%, respectively, higher than media-treated plants (Table 1). However, PGPR addition reduced P concentration in roots of P0 plants by 17% and young leaves of P1 plants by 19%. Nevertheless, PGPR inoculation tended to increase root P uptake by 28% although there was no effect on root specific P uptake compared with sterile media addition under P0. TAA decreased by 40% in young leaves and increased by 78% in roots by PGPR under P0. PGPR also increased root TAA under P1 by 3-fold (Table 2). These results were similar to our pre-experiment, and they indicate an improvement in plant growth by PGPR inoculation under P0 with an addition of culture media; however, PGPR also altered amino acid biosynthesis in plant roots at both P0 and P1 levels.

### 2.6. Root-Associated Bacterial Community

**Figure 6:**
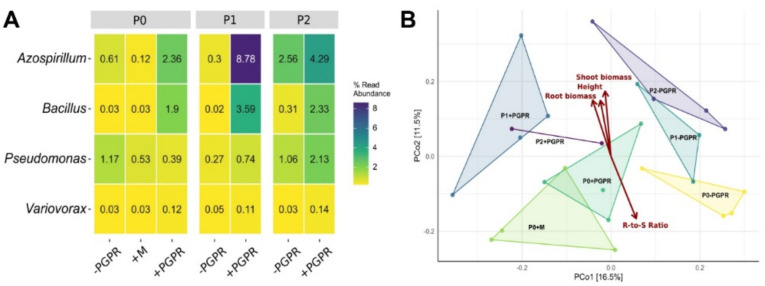
(**A**) Bacterial community composition at genus level detected in and on the root tissue of PGPR-inoculated and non-inoculated plants at P0, P1, and P2, and (**B**) their correlation with plant growth parameters. The correlation between bacterial community composition and plant growth was performed with principal coordinates analysis (PCoA) using Bray–Curtis distance units. The correlations are significant at *p* = 0.009, *p* = 0.003, *p* = 0.001, and *p* = 0.001 for plant height, root biomass, shoot biomass, and root-to-shoot ratio, respectively. The analysis was based on four biological replications (*n* = 4), except for P2 + PGPR with *n* = 2 and P1-PGPR with *n* = 3 due to insufficient sample material.

To gain a better understanding of how PGPR co-inoculation interacted with plants, the root-associated bacterial community was also determined by amplicon-based 16S rRNA gene analysis. Overall, 1,092,304 raw reads were produced, of which 820,818 remained after quality filtering, and chimera removal. Within the obtained dataset, the 25 most abundant root-associated bacterial orders are shown in Figure S2. Furthermore, it was possible to classify the reads partially at genus or species level. Thus, three of the five inoculated PGPR strains on the species level and four of them at the genus level were identified within the dataset, as shown in Figure 6A. In addition, Faith’s phylogenetic diversity (PD) was lowest in the P0 supplemented with sterile media (41.3), and highest in P1 and P2 without PGPR addition (45.5 and 45.7), while the treatments with PGPR addition had a similar PD (44.3, 44.9, and 44.5). The principal coordinates analysis (PCoA) revealed a significant correlation of the bacterial community composition with plant height, root and shoot biomass, as well as the root-to-shoot ratio (Figure 6B). The bacterial communities of P treatments without PGPR addition cluster together and separately from those of the P treatments with PGPR as well as sterile media addition. This indicates differences in bacterial community composition between PGPR-treated and untreated roots, independent from the impact of P fertilization on the community (Figure S2).

## 3. Discussion

### 3.1. P Deficiency and Toxicity Conditions for Potato

Understanding the morphophysiological and metabolic responses to P deficiency and toxicity conditions is important to understand how potato plants use P when it is either in deficit or in high abundance. Characterization of plant responses to excess P supply for potato has not been elucidated yet. Results of the present study show P deficiency at P0 and P toxicity at P30 and P40, and P optimum at P5. Huett et al. [21] also reported P deficiency and adequate P supply if the P concentration in leaf tissue is below 2.3 mg g^−1^ and between 3.0–6.0 mg g^−1^, respectively. Therefore, potato plants grown under P1 and P2 were also within the range of sufficient P supply. The survival, biomass production, and P accumulation of plants under the condition without P (P0) in the present study was due to the carry-over effect of P during seedling germination. In contrast, Fernandes and Soratto [11] and Barben et al. [7] observed P deficiency symptoms at 1 mg P L^−1^ in nutrient solution. This difference may be due to the use of different cultivars. Nevertheless, the definitions of P optimum (5 mg L^−1^) and P toxicity (≥30 mg L^−1^) are comparable with that of Fernandes and Soratto [11] and Barben et al. [7]. This result is also in agreement with the result of Bhatti and Loneragan [22], who reported P toxicity in wheat for the first time when leaf P concentration exceeded 10 mg g^−1^ of dry matter.

### 3.2. Effect of P Deficiency and Toxicity on Plant Growth and Nutrient Assimilation

The decreased leaf number under P deficiency implied that leaf initiation rates and shoot apical meristem activity were inhibited, which subsequently reduced cell expansion and individual leaf size [23]. Although plant height and leaf number under P toxicity were not affected, leaf expansion could be restricted, causing reduced shoot biomass. Therefore, less expanded leaves of plants under P deficiency and toxicity resulted in a relative increase of the chlorophyll concentration. Similarly reported by Mengel et al. [24], the leaves of P-deficient plants eventually become thick and appear to be bluish green. Under P deficiency, impaired shoot growth could be caused by the preferential assimilate distribution to roots for improved P uptake, causing a high root-to-shoot ratio [9]. In this situation, plants require a P conservation strategy through the mobilization of P from old leaves to young leaves and roots [25]. Consequently, old leaves are shed off (Figure 1A). In contrast to P deficiency, root biomass is reduced at a greater magnitude than shoot biomass under P toxicity (Figure 1D). The reduction of root growth under P toxicity could be due to less availability of P in the roots. The reduced P could be linked to a reduction in phosphate uptake activities under excess P application to prevent the accumulation of toxic P concentration in the roots while the phosphate mobilization from roots to shoots still occurs [25,26]. Consequently, P concentration in young leaves, stem, and old leaves is less affected. A further experiment may be required to elucidate P transporter genes in various parts of plant.

The pH range 5.5–6.5 of nutrient solution in the present study is optimal for the availability of nutrients [27]; therefore, the uptake of other minerals by plants is solely affected by P availability in the nutrient solution. In line with the previous report by Pang et al. [28] on legumes, we found an increased N concentration in leaves under P0, followed by a substantial decrease until P12, but recovery at increased P application to P30 and P40 (Figure 2B). This could explain the results with respect to the relative increase of leaf chlorophyll (Figure 1E), which is approximately proportional to leaf N under P limiting condition [29]. The accumulation of N under P deficiency and toxicity could be an adaptive response to meet the N demand for protein synthesis under stress conditions [30]. The variations in tissue N concentrations under varying P supply also, subsequently, influenced the C-to-N ratio (Figure 2D), which indicates stolon elongation and tuber yield formation [31]. The high concentrations of Mg, Fe, Mn, and Zn under P deficiency (Figure 2E,G–I) imply that the uptake and use of these minerals were altered. Owing to the roles of Mg in photosynthesis and assimilate transport [32], plants need a huge amount of this element for P and carbohydrate translocation under P deprivation. The increased uptake of metallic ions such as Fe and Zn under P deficiency could be a plant strategy to decrease their amounts in the rhizosphere in order to reduce the complex formation between phosphate and these ions [33]. Another explanation for elevated Fe and Zn in roots could be due to the increased availability of these minerals in the absence of P in the nutrient solution. In Arabidopsis, Misson et al. [30] showed a suppression of iron transporter *IRT1* in roots under P deficiency which is a mechanism in maintenance of Fe homeostasis. Moreover, excess P supply also resulted in the accumulation of Fe in roots (Figure 2G), which could react with hydrogen peroxide in plants to produce hydroxyl radicals, leading to the death of meristematic cells and causing root biomass reduction [26]. It has been shown that an excess supply of P reduces Zn and Ca availability near the root surface, preventing its uptake and translocation to the shoots [1]. In the present study a similar tendency was observed, but leaf Zn concentration was still in a sufficient range (>0.015 mg g^−1^; Huett et al. [21]). Therefore, P-induced Zn deficiency does not account for P toxicity.

### 3.3. Effect of P Deficiency and Toxicity on Plant Secondary Metabolite and Root Metabolite Profiling

Plants use various adaptation strategies in response to stress induced by P deficiency and toxicity. Under P deficiency, the accumulation of secondary metabolites and free amino acids in potato plants ranged from 60% to 200% higher than those under optimal P conditions (Figure 3). The previously reported accumulation of total flavonoids in tomato and Arabidopsis [12], leaf anthocyanins in sunflower [34], and total free amino acids in barley [35] under P deficiency are also within this range. However, Aleksza et al. [14] found 3.5-fold increase of proline in Arabidopsis without P application. The variation of the results may be caused by different plant species and experimental conditions. Moreover, there was also an accumulation of these compounds at different magnitudes under P toxicity. The analyses were conducted using oven-dried (60 °C) samples, but this sample-processing method did not significantly influence the results based on a pre-test by comparing oven- and freeze-dried samples (Table S2), which was also in agreement with Volf et al. [36]. Fini et al. [37] reported a disruption of photosynthetic apparatus under stress that could be induced by P deficiency or toxicity, leading to increased sensitivity of plant to high light. Therefore, the up-regulation of total flavonoids and total anthocyanins in leaves occurs as a protection mechanism against light-induced oxidative damage under P deficiency and toxicity stress [37]. The increase of leaf anthocyanins under P toxicity was also reported in Arabidopsis [26]. Proline also acts as a stress indicator, leading to further increase of stress-related metabolites. In Arabidopsis, the accumulation of proline under P starvation is mediated by stress-induced abscisic acid, and it is also caused by the up-regulation of *P5CS1* and *PDH2* genes, which control proline metabolism [14]. The mechanism behind proline accumulation and P toxicity has not been explained yet; however, proline in roots probably acts to scavenge hydroxyl radicals [38], which are produced under elevated Fe conditions (Figure 2G).

In addition to the secondary metabolite quantification, a comprehensive metabolite profiling of roots provided us valuable information about how primary and secondary metabolites change in response to P deficiency and toxicity (Figure 4). Sugars decreased in roots under P deficiency at a greater scale than those under P toxicity. Under P deficiency, low cytosolic P results in a reduced phosphate release from the Calvin cycle for sucrose synthesis [10]. Owing to high leaf and root Mg, carbohydrate transport from leaves to roots may not be inhibited under P deficiency [32,39]. Therefore, the reduction of leaf sucrose leads to a decrease in reducing sugars and their concentrations in roots. A strong reduction in glucose under P deficiency results in a pyruvate decrease. However, high cytosolic P under P toxicity alters sugar metabolism due to over-expression of hexokinase activity under elevated ATP levels [40]. Consequently, hexose sugars (glucose, fructose, and mannose) may be rapidly converted to their phosphorylated forms, resulting in a low hexose sugar concentration in roots under P toxicity condition. If this hypothesis is true, improving phosphorylated sugars in roots is important for the biosynthesis of essential molecules such as amino acids [15].

The levels of aromatic amino acids (tyrosine and phenylalanine) were enhanced under P deficiency. P toxicity condition also tended to increase the concentration of these aromatic amino acids (Figure 4). The increase in the amount of phenylalanine and tyrosine in roots was consistent with the accumulation of total flavonoids in roots under both P deficiency and toxicity. This is probably due to the enhanced phenylalanine/tyrosine ammonia lyase activity under stress conditions to release nitrogen for phenylalanine and tyrosine metabolism, which contribute to protein synthesis. Meanwhile, the carbon products are transferred into flavonoid biosynthesis pathway via 4-coumaroyl-Coenzyme A [12]. The high activity of phenylalanine ammonia lyase was also reported on maize roots under P deficiency [41].

P deficiency and toxicity also reduced the production of most of the organic acids (i.e., succinate, fumarate, and malate) in the TCA cycle. The reduction in α-ketoglutarate was observed only under P deficiency. This is similar to the results of Huang et al. [42] on barley roots under deficit P supply. However, Müller et al. [43] reported an enhanced organic acid concentration in lupin roots, whereas Nguyen et al. [15] found no change in organic acid concentrations in maize roots under P deficiency. These differences could be caused by plant species, cultivation systems (pot vs. hydroponic condition), and harvesting stage. The shortage of carbohydrates under P deficiency could be responsible for the reduced levels of organic acids in roots. For instance, fumarate represents a great percentage of fixed carbon; therefore, photosynthesis impairment induced by P deficiency results in low carbon fixation in leaves and its translocation into the roots, causing low fumarate in roots [30,44]. Moreover, organic acids might have been secreted into the nutrient solution in response to P starvation [9]; thus, their concentrations in roots remain low. The mechanisms for the reduction in the levels of organic acids under P toxicity are not clear; however, it could also be related to the shortage of carbohydrate. Another explanation for the low organic acid concentrations in roots could be their high metabolism under stress conditions induced by both P deficiency and toxicity. For instance, α-ketoglutarate is the precursor for γ-aminobutyrate (GABA), which is produced for plant abiotic stress protection [45]. Moreover, GABA can also be formed from proline by nonenzymatic pathways [38], explaining the consistently high proline and GABA concentrations under both P deficiency and toxicity.

In line with the increased levels of amino acids in barley and lupin under P starvation [42,43], P deficiency in the present study enhanced the concentrations of most amino acids in the roots. Of all the measured amino acids, aspartate plays an important role in the biosynthesis of other amino acids such as asparagine, threonine, lysine, and isoleucine [15]. Therefore, the accumulation of aspartate results in increased concentration of other amino acids in roots. Genes responsible for aspartate family pathways are stimulated under stress conditions due to carbohydrate deprivation [46]. Moreover, the up-regulation of glutamine in P-deficient roots can be an adaptive response related to N concentration for protein synthesis because glutamine plays an indispensable role as amino donor in the biosynthesis of amino acids and N-containing compounds [30]. However, P toxicity increased the levels of amino acids to a lesser extent, which is evident only for alanine and valine. Their accumulation is a protective mechanism of plants in response to stress induced by P toxicity. For instance, alanine is a well-known stress-responsive amino acid, and its metabolism is activated under various stress conditions [46]. Therefore, higher amino acid concentrations in the roots may lead to potato plants being more tolerant of P deficiency and toxicity.

### 3.4. Plant Growth Promotion by PGPR under Low P Conditions

The co-inoculation of five PGPR strains improved plant biomass, total root length, root surface area, and plant secondary metabolites under P0 (Table 1 and Table 2). The in-vitro determination of IAA from the bacterial cultures revealed a substantial IAA production by each strain. Even though phytohormones in the roots were not quantified in this study, bacterially derived IAA may also contribute to root growth and, subsequently, shoot biomass enhancement. There have been reports of the production of IAA and other phytohormones by *Variovorax paradoxus* [17], *Azoarcus sp.* [47], *Azospirillum sp*. [48], *Bacillus subtilis* [49], and *Pseudomonas putida* [50]. Most of these reports have shown the role of IAA in root system development. Present in a low concentration, IAA can stimulate primary root growth, whereas high IAA concentrations enhance root hairs and lateral roots [16]. Consequently, total root length and root surface area are improved to scavenge limited amount of P in the nutrient solution. Consequently, root P uptake was enhanced. These results also suggest a further study on root phytohormone determination which could be influenced by low P availability and PGPR inoculation. In the present study, the addition of the bacterial culture media increased P concentration of the nutrient solution by 0.5 mg P L^−1^. This low amount of P can be beneficial for plants through improved P uptake by root length elongation in PGPR-inoculated plants. The further addition of P might have a minor or even no impact, as PGPR also need P for their growth, survival, and functioning [51]. However, the low root P concentration of PGPR-treated plants may be due to the dilution effect. The TAA in roots was also altered by PGPR. Mhlongo et al. [52] reported the regulation of aromatic amino acids in tomato plant roots following *Pseudomonas sp.* inoculation. These amino acids are important for secondary metabolite biosynthesis in connection with cell wall lignification and chemical defense against pathogens. Nevertheless, an amino acid such as tryptophan is the precursor of IAA, which is a plant defense metabolite and also facilitates plant root elongation [16,53]. The minor response of PGPR under P1 and P2 could be caused by less reliance of plants on PGPR to improve root growth and nutrient uptake. Based on the results described above, P1 and P2 might be already sufficient for plant growth and development. Plant-microbial symbiosis is a carbon-expensive process; therefore, plants try to make cost–benefit analyses for the efficient use of their carbohydrates [54].

Additionally, determining the root-associated bacterial community composition provides further insight into PGPR–plant interactions. The results show the identification of three of the added species in the derived dataset on species level and four of them at the genus as well as at the family level, indicating an attachment to—or even a penetration of—the roots, as well as a survival of the added PGPR. The absence of *Azoarcus* could be explained by a missing root association and/or competition between all PGPR. Furthermore, PD hints at a stabilization of the bacterial community and diversity by PGPR addition. The PD for treatments with PGRP addition is stable across the different P levels, suggesting that the influence of PGPR is greater than that of the P addition. Compared to P0 without PGPR inoculation, the PD is increased for P1 and P2 and decreased for P0 + M. The bacterial community composition is affected by PGPR and P addition which is also illustrated by the topology of the PCoA. Overall, the PCoA revealed a significant correlation of the bacterial community composition with plant height, root and shoot biomass, and the root-to-shoot ratio, indicating an effect of the media addition, but also a stronger and more pronounced effect of the PGPR addition on the bacterial community. A positive effect of PGPR addition on plant development and the composition of rhizosphere bacterial communities has also been shown by e.g., Chen et al. [55] and Pereira et al. [56]. Nevertheless, the root-associated microbial community composition was also affected by the P addition, which has also been shown for the rhizosphere inhabiting microbial community of blueberries [57].

## 4. Materials and Methods

### 4.1. Experiment Setup and Plant Cultivation

**Figure 7:**
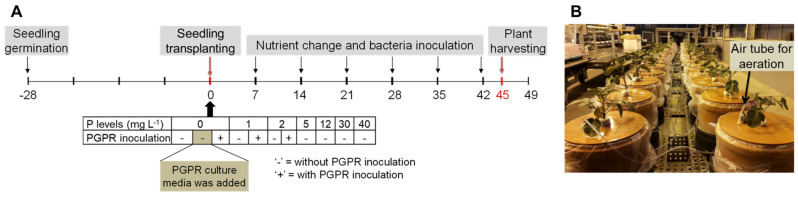
(**A**) Experimental design and timeline and (**B**) growing condition and pot arrangement in the greenhouse.

The hydroponic experiment was conducted under greenhouse conditions. External light was supplied using sodium vapor lamps with photosynthetic photon flux density (PPFD) of 400 µmol m^−2^ s^−1^ at plant level from 6 a.m. to 10 p.m. The average temperature during the growing period was 19.3 °C ± 5.0 °C, and the average relative humidity was 42.8% ± 9.9%. The experiment was arranged in a randomized complete block design under different P levels and PGPR inoculation scenarios, and it had four replications. The P levels were 0, 1, 2, 5, 12, 30, and 40 mg P L^−1^ with the use of Ca(H_2_PO_4_)_2_ × H_2_O, and these P treatments are referred as P0, P1, P2, P5, P12, P30, and P40, respectively, in the entire manuscript. To assess the effects of PGPR, the low P levels (P0, P1, and P2) were treated with and without PGPR inoculation (Figure 7A).

Seedlings of the potato cultivar “Milva” (Europlant Pflanzenzucht, Lüneburg, Germany) were raised in a pot (one seedling per pot) filled with 3 kg of quartz sand, which received 8 mg P kg^−1^. Other nutrients were applied at optimum (Table S3). Four weeks later, the healthy and uniformly grown seedlings were removed from quartz sand and transplanted in 6 L pots (one plant per pot), which contained nutrient solution with different P concentrations. Other nutrients were applied at sufficient amounts (Table S4). The nutrient solution had a pH of 5.5–6.5. During the first two days after seedling transplanting in nutrient solution (DAT), the nutrients were supplied at 20% of the full concentration, and it increased it to 50%, 70%, and 100% every two days. The nutrient solution was constantly aerated (Figure 7B). The nutrient solution was renewed once per week until the end of the experiment.

### 4.2. Bacteria Culture and Inoculation

Five taxonomically diverse PGPR including *Variovorax paradoxus* DSM 30,034 (NBRC 15149), *Azoarcus sp.* DSM 9506, *Azospirillum sp.* DSM 1842, *Bacillus subtilis* DSM 21393, and *Pseudomonas putida* DSM 6125 (KT2440) were obtained from German Collection of Microorganism and Cell Culture (DSMZ), Braunschweig. The biome of each bacterium is listed in Table 3. The bacteria cultivation procedures are depicted in Figure S3. For inoculation, a 50 mL mixture of the five bacteria cultures was prepared by adjusting the density of each strain based on optical density measurement at 600 nm (OD_600_). The mixture, which contained 2.8–10 × 10^9^ colony-forming units (CFU) per ml of each strain, was then used to inoculate the nutrient solution at 7 DAT. A control treatment under P0 was also established by adding sterile media (Figure 7A, highlighted in ochre). Both bacteria mixture and sterile media were renewed once a week after the exchange of nutrient solution.

### 4.3. Quantification of Bacteria Derived Indole-3-Acetic acid

Each of the strain cultures was diluted to 0.4 at OD600. A total of 50 mL of the diluted culture was centrifuged at 2700× *g* for 15 min, to collect the supernatant for colorimetric quantification of indole-3-acetic acid based on Sarwar and Kremer [61]. A total of 150 µL of culture supernatant was added to 100 µL of Salkowski reagent (34.3% of HClO_4_ and 10 mM of FeCl_3_). Fresh, sterile media for each bacterium were used as blanks. The mixture was incubated for 30 min at room temperature and read in a plate reader (Biotek, Bad Friedrichshall, Germany) at 530 nm absorbance.

### 4.4. Plant Growth Measurements, Harvest, and Sample Processing

Plant height was measured at 11, 18, 28, and 45 DAT. At 18 and 45 DAT, the number of leaves was also counted. Whole plants were harvested at 45 DAT and separated into shoots (aerial parts of plants) and roots. The roots were washed with distilled water three times and dried with tissue paper. The shoots were further separated into young leaves, old leaves, and main stem for sample preparation and analyses. Young leaves were defined as petioles at the third and fourth position from the top, and old leaves as petioles at the first and second position from the bottom with less than 30% chlorosis and necrosis. Side shoots and remaining leaves were not included in sample preparation, but they were included for shoot biomass calculation. All samples were then oven-dried at 60 °C for 72 h. A part of the root samples was immediately frozen in liquid nitrogen and afterward freeze-dried for four days using an EPSILON 2-40 freeze dryer (Christ, Osterode am Harz, Germany). After that, all samples were milled (DFH 48 Culatti, Kinematica, Malters, Switzerland) to obtain a fine power (<0.5 mm).

To quantify the effect of PGPR on root growth, one third of the fresh root sample of PGPR-treated and non-treated plants of the same P level was scanned for total root length and root surface area. Each sample was split into subsamples of 5–10 root segments. To minimize the overlapping, root segments of each subsample were separated and placed in a transparent water bath (20 × 24 × 2 cm) containing 1 cm of water. A digital image of each subsample was acquired using an EPSON Perfection V800 Photo scanner (Epson, München, Germany) and analyzed using WinRHIZO image analysis software (Regent Instruments, Québec city, QC, Canada).

### 4.5. Mineral Nutrient Analyses

Based on the method described by Koch et al. [32], the concentrations of P, potassium (K), Ca, Mg, sulfur (S), Fe, Mn, and Zn were determined in young leaves, old leaves, stem, and roots. About 3.5–4 mg of each sample was also weighed in a 5 × 9 mm tin capsule (IVA Analysentechnik, Meerbusch, Germany) and analyzed for N and C concentration against acetanilide standard by a using dry combustion method in a Vario EL analyzer (Elementar, Langenselbold, Germany).

### 4.6. Chlorophyll, Proline, and Total Free Amino Acid Analyses

A total of 20 mg of each oven-dried young leaf, old leaf, and root samples was mixed with 250 µL of 80% ethanol for 30 min at 95 °C. It was then centrifuged at 10,600× *g* for 10 min to collect the supernatant. The procedure was repeated with 150 µL of 80% ethanol and then three times more with 250 µL of 50% ethanol. The supernatants from all steps were combined. For analyses, the chlorophyll concentration of young leaves was photometrically determined based on Koch et al. [32]. Proline concentration was measured according to Bates et al. [62]. Total free amino acid concentration was determined based on the reaction of amino acids with fluorescamine. A total of 2 µL of the extract or glutamic acid standard was mixed with 15 µL of sodium borate buffer (0.1 M; pH 8.0), 90 µL of fluorescamine (0.1% in acetonitrile), and 10 µL of water. After incubation for 5 min, the fluorescence was measured in a plate reader at 360/40 nm excitation and 460/40 nm emission.

### 4.7. Secondary Metabolite Analyses

A total of 50 mg of oven-dried young leaf, old leaf, and root sample was extracted twice with 1 mL of 60% methanol containing 1% HCl. The supernatant was collected and filled up to 2 mL with the acidified methanol. For measurements, total anthocyanins in young leaves were determined spectrophotometrically, following Hada et al. [63], using Cyanidin-3-glucoside as standard. The AlCl3 colorimetric method of Chandra et al. [64] was adapted to determine total flavonoid concentration in the plant material using Quercetin-3-glucoside standard in a plate reader. Total phenolic concentration was determined using the Folin–Ciocalteu assay. A total of 300 µL of the extract was mixed with 1 mL of 0.5 M NaOH, 2.6 mL of water, and 100 µL of the Folin–Ciocalteu reagent, and the mixture was incubated for 15 min at 37 °C. After cooling, the total phenolic concentration was measured in a UV-Vis spectrophotometer (Hewlett Packard, Hamburg, Germany) at 736 nm absorbance. Gallic acid was used as standard.

### 4.8. Root Metabolite Profiling

The metabolite extraction and analytical procedures were conducted at Lifespin GmbH, Regensburg, Germany. Metabolites in roots were extracted by homogenizing 100 mg of freeze-dried samples with 1.5 mL of 1 M phosphate buffer (pH 6.8) containing 5% D2O and 0.01% NaN_3_. The mixture was incubated for 20 min at 85 °C and cooled down for 40 min to room temperature. The supernatant was collected after 10 min centrifugation at 10,600× *g*. Then 630 µL of the aliquot was suspended with 70 µL of additive solution (Lifespin, Regensburg, Germany) containing internal reference standards. A total of 600 µL of the mixture was afterward filled in a 5 mm NMR-tube and analyzed by nuclear magnetic resonance (NMR) spectroscopy using a Bruker AVANCE III HD 600 MHz spectrometer (1D 1H noesygppr NMR spectrum, NS = 32, T = 298K). The obtained spectra were analyzed with Lifespin’s proprietary profiling software (V1.2.3, customized for potato root extracts). The metabolite was identified by comparison with Lifespin’s database and subsequently quantified against pyrazine (8.66 ppm, 4 protons). The concentration of each metabolite was expressed as mg per g dry matter (mg g^−1^).

### 4.9. Root-Associated Bacterial Community

#### 4.9.1. DNA Isolation and 16S rRNA Gene Amplification

To analyze the root-associated bacterial community, DNA was extracted from 500 mg freeze-dried root samples using the DNeasy PowerSoil Kit (Qiagen, Hilden, Germany) as recommended by the manufacturer. The obtained DNA was used for amplification of the V3–V4 region of the 16S rRNA gene. The bacterial primer pair S-D-Bact-0341-b-S-17 and S-D-Bact-0785-a-A-21 targeting the V3–V4 region was used as described by Klindworth et al. [65] with addition of adapters for Illumina MiSeq sequencing. The PCR reaction mixture contained 5-fold Phusion GC buffer, 200 µM of each of the four deoxynucleoside triphosphates, 5% DMSO, 0.4 µM of each primer, 1 U of Phusion HF DNA polymerase (Fisher Scientific, Schwerte, Germany), and 25 ng of DNA as template. The cycling scheme used for DNA amplification was as follows: initial denaturation at 98 °C for 5 min and 25 cycles of denaturation at 98 °C for 45 s, annealing at 60 °C for 30 s and extension at 72 °C for 30 s, followed by a final extension at 72 °C for 10 min. PCR reactions were performed in triplicate for each sample. The resulting PCR products were pooled equimolar and were purified using the GeneRead Size Selection kit (Qiagen, Hilden, Germany) as recommended by the manufacturer. The PCR products were quantified using the Quant-iT dsDNA HS assay kit and a Qubit fluorometer, as described by the manufacturer (Invitrogen, Schwerte, Germany). Indexing of the PCR products was performed by the Göttingen Genomics Lab (Göttingen, Germany) using the Nextera XT Index kit as recommended by the supplier (Illumina, San Diego, CA, USA). Sequencing of 16S rRNA genes was performed using the dual index paired-end approach (2 × 300 bp) with v3 chemistry for the Illumina MiSeq platform as recommended by the manufacturer (Illumina, San Diego, CA, USA).

#### 4.9.2. Sequence Data Processing and Analyses

Adapter removal and quality filtering of raw paired-end sequences was done using fastp v0.19.6 [66], with base correction in overlapping regions, a Phred quality score of 20, size filtering of sequences longer 50 bp and per read trimming by quality with sliding windows of 4 and soft-clipping. The quality-filtered paired-end reads were merged by PEAR v0.9.11 with default parameters [67]. Primer removal was conducted using cutadapt v1.18 [68]. Subsequently, dereplication, denoising, as well as chimera detection and removal (de novo followed by reference-based) was performed with VSEARCH v2.13.0 [69]. Finally, the raw reads were mapped to amplicon sequence variants (ASVs). The ASVs were taxonomically classified with BLAST+ v2.7.1 against the SILVA 132 SSU reference database [70]. Subsequently, extrinsic domain ASVs and chloroplasts were removed from the dataset. Sample comparisons were performed at the same surveying effort of 8333 sequences per sample. Statistical analyses were done using the ASV table in R version 3.5.3 [71]. Species richness, alpha diversity estimates, and rarefaction curves were determined using *ampvis2* v2.4.7 [72]. To analyze the root-associated microbial communities in the differently treated plants, PCoA based on Bray–Curtis distance measures was performed without data transformation [73]. The correlation of environmental parameters to the bacterial community composition was performed using the *envfit* function of the *vegan* package version 2.5–4 [74] and projected into the ordination with arrows with a *p*-value cutoff of ≤0.05.

### 4.10. Statistical Analysis

Data of plant growth, mineral concentration, secondary metabolites, and metabolite profiles were subjected to an analysis of variance (ANOVA). Tukey’s HSD test at *p* ≤ 0.05 was conducted for pairwise comparison of the treatments when there were significant differences of ANOVA. Contrast analyses were performed to answer relevant questions regarding different effects including the effect of P, the effect of PGPR at low P levels (P0–P2), and the effect of bacteria by excluding culture media (P0 + PGPR vs. P0 + M). These analyses were performed with Statistix 8.0 (Analytical Software, Tallahassee, FL, USA).

## 5. Conclusions

**Figure 8:**
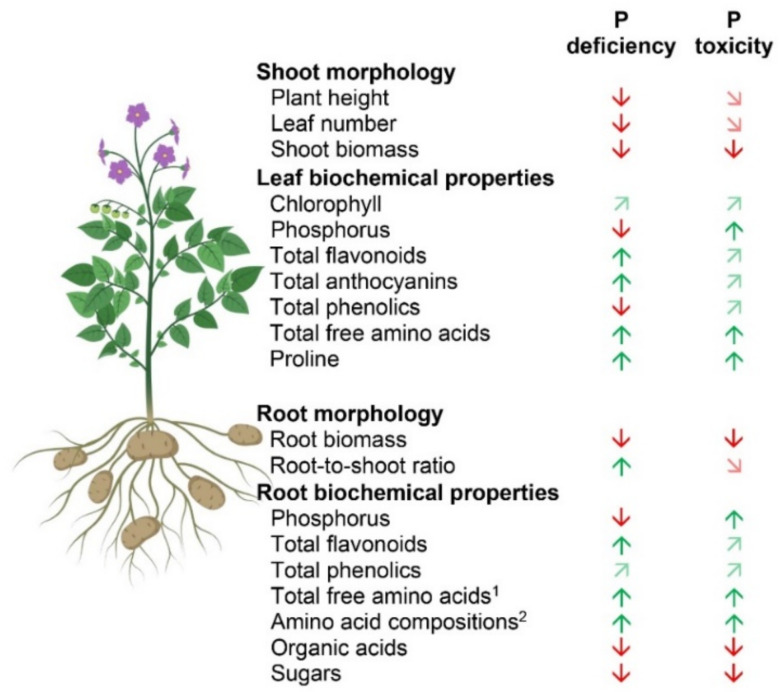
Summary of the morphological and biochemical responses of the potato plants to deficient and toxic P conditions. Green and red arrows indicate significant increase and decrease (*p* ≤ 0.05), respectively, compared to optimum P condition. Light green and light red diagonal arrows indicate a tendency of increase and decrease, respectively. ^1^ based on colorimetric determination; ^2^ based on nuclear magnetic resonance (NMR) spectroscopy determination. Figure 8 was created using BioRender (https://biorender.com, accessed on 14 December 2020) as part of Academic License.

**Figure 9:**
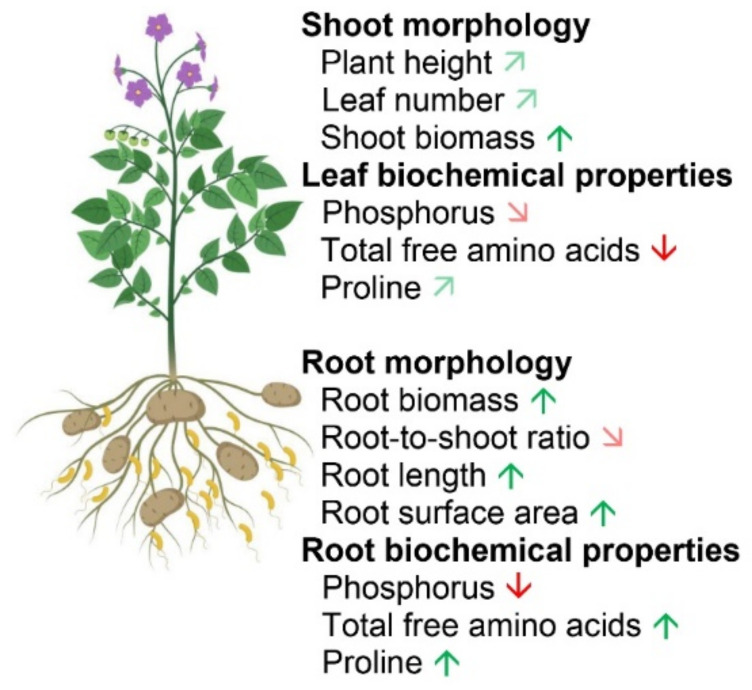
Summary of the morphological and biochemical responses of the potato plants to PGPR inoculation under P deficiency. Green and red arrows indicate significant increase and decrease (*p* ≤ 0.05), respectively, compared to optimum P condition. Light green and light red diagonal arrows indicate a tendency of increase and decrease, respectively. Figure 9 was created using BioRender (https://biorender.com, accessed on 14 December 2020) as part of Academic License.

The results of the present study contribute to a deeper understanding of P deficiency and toxicity-adaptive responses in potato plants. In addition to the previous reports on P deficiency in potato plants, further insights into nutrient interactions and metabolite changes were elucidated, which are closely related to tolerance mechanisms under both P deficiency and toxicity. The morphological and biochemical changes under P deficiency and toxicity are summarized in Figure 8. There was a stronger switch from primary to secondary metabolism under P deficiency compared to those under P toxicity. Moreover, PGPR could increase plant tolerance under P deficiency through their contribution to root growth and plant biomass improvement (Figure 9). The root-associated microbial communities showed an impact of the PGPR addition on the community composition; they also revealed a significant correlation with plant height and root as well as shoot biomass. Although the potato plant responses to P deficiency and toxicity condition was characterized, further studies may be required to test the specificity of these responses by other cultivars. The positive effects of PGPR co-inoculation also provide an outlook to assess the effect of individual strains on plant biomass, P uptake, and secondary metabolites of potatoes.

## Figures and Tables

**Table 1:** Effect of PGPR on shoot and root growth at low P applications.

P level (mg L^−1^)	PGPR	Height (cm)	Leaf Number (plant^−1^)	Root (g plant^−1^)	Shoot (g plant^−1^)	R-to-S Ratio	Total Root Length (10^4^ cm)	Root Surface Area (10^3^ cm^2^)
0	−PGPR	13.3 ± 0.5 b	4.3 ± 0.3 b	8.3 ± 0.7 c	3.4 ± 0.4 c	2.6 ± 0.4	n.d.	n.d.
	+M	20.5 ± 0.9 a	14.3 ± 1.4 a	52.0 ± 3.3 b	26.3 ± 0.7 b	2.0 ± 0.1	4.7 ± 0.0 b	2.8 ± 0.0 b
	+PGPR	20.6 ± 0.9 a	15.3 ± 0.5 a	76.1 ± 2.6 a	35.1 ± 2.2 a	2.2 ± 0.2	6.8 ± 1.0 a	3.7 ± 0.4 a
1	−PGPR	35.3 ± 1.1	17.3 ± 0.3	92.3 ± 4.3	102.2 ± 4.7	0.9 ± 0.0	6.6 ± 0.0	3.6 ± 0.0
	+PGPR	32.8 ± 2.1	17.3 ± 1.3	79.9 ± 6.8	103.9 ± 7.8	0.8 ± 0.0	6.2 ± 0.5	3.5 ± 0.2
2	−PGPR	37.0 ± 1.2	18.0 ± 1.0	97.9 ± 6.3	125.2 ± 7.4	0.8 ± 0.1	7.6 ± 0.5	4.3 ± 0.3
	+PGPR	38.0 ± 1.6	17.0 ± 0.9	85.7 ± 7.0	115.6 ± 10.8	0.8 ± 0.1	6.4 ± 0.2	3.4 ± 0.1

Mean ± SE with different letters in the same column and same P level are significantly different at *p* ≤ 0.05 by Tukey’s HSD test. No indication means non-significant difference. M = sterile culture media, n.d. = not determined due to insufficient sample material.

**Table 2:** Effect of PGPR on P, root P uptake, specific P uptake, total free amino acids (TAA), and proline concentrations in young leaves and roots at low P applications.

P Level (mg L^−1^)	PGPR	P (mg g^−1^)		Root P Uptake (mg plant^−1^)	Specific P Uptake (µg P cm^−2^)	TAA (mg g^−1^)		Proline (µmol g^−1^)	
		Leaves	Roots			Leaves	Roots	Leaves	Roots
0	−PGPR	1.7 ± 0.2 b	2.1 ± 0.1 a	1.1 ± 0.1 b	n.d.	79.0 ± 8.3 a	71.6 ± 7.8 a	30.2 ± 2.6	5.7 ± 0.3
	+M	2.4 ± 0.1 a	2.3 ± 0.1 a	7.2 ± 0.4 a	2.6 ± 0.2	90.1 ± 3.9 a	37.6 ± 4.1 b	23.1 ± 1.1	6.3 ± 0.8
	+PGPR	2.3 ± 0.1 a	1.9 ± 0.1 b	9.2 ± 0.9 a	2.7 ± 0.6	54.0 ± 8.7 b	67.0 ± 9.3 a	23.2 ± 1.8	8.0 ± 0.5
1	−PGPR	4.3 ± 0.1 a	2.7 ± 0.2	15.6 ± 1.3	3.9 ± 0.3	58.3 ± 3.9	24.5 ± 0.7 b	20.7 ± 1.4	6.5 ± 1.0
	+PGPR	3.5 ± 0.1 b	2.5 ± 0.2	12.4 ± 1.0	3.6 ± 0.3	58.2 ± 3.3	82.4 ± 2.3 a	20.5 ± 1.1	6.5 ± 0.0
2	−PGPR	4.9 ± 0.3	3.6 ± 0.4	22.7 ± 4.1	4.4 ± 0.5	57.5 ± 12.5	32.1 ± 3.3	16.9 ± 0.4	4.5 ± 0.6
	+PGPR	4.6 ± 0.2	3.8 ± 0.2	20.3 ± 1.8	5.5 ± 0.6	42.5 ± 4.4	45.1 ± 7.7	19.7 ± 0.6	5.1 ± 0.3

Mean ± SE with different letters in the same column and same P level are significantly different at *p* ≤ 0.05 by Tukey’s HSD test. No indication means non-significant difference. M = sterile culture media, n.d. = not determined due to insufficient sample material.

**Table 3:** List of the bacteria strains, biome, and their growth-promoting effects.

Bacterium	DSMZ^#^ Code	Biome	Plant Growth-Promoting Effects
*Variovorax paradoxus*	DSM 30034	Soil	Rhizosphere and endosphere bacterium of potato [58,59] Producing indole-3-acetic acid (IAA); improving plant biomass and P uptake of pea [17]
*Azoarcus sp.*	DSM 9506	Laboratory aquifer column	Endosphere bacterium, producing IAA, fixing N_2_, and enhancing P uptake of rice [47]
*Azospirillum sp.*	DSM 1842	Maize roots	Endosphere bacterium and IAA synthesis in rice [48] Producing IAA and promoting plant biomass of potato [18]
*Bacillus subtilis*	DSM 21393	Potato tubers	Endosphere bacterium maize [60] Producing IAA; improving root length and plant biomass of potato [49]
*Pseudomonas putida*	DSM 6125	Unknown	Endosphere bacterium of potato [19] Producing IAA, improving root growth of canola [50]

^#^ DSMZ = German Collection of Microorganism and Cell Culture (Braunschweig, Germany).

## Data Availability

The 16S rRNA gene sequences were deposited in the National Centre for Biotechnology Information (NCBI) Sequence Read Archive (SRA) under BioProject number PRJNA680693 and SRA accession numbers SRR13132005 to SRR13132029. Data from measurements are available upon request from the corresponding author.

## Supplementary Materials

The following are available online at https://www.mdpi.com/article/10.3390/ijms22105162/s1.
