# Supplementary material for: Morphological and Metabolite Responses of Potatoes under Various Phosphorus Levels and Their Amelioration by Plant Growth-Promoting Rhizobacteria
Source: Int J Mol Sci. 2021 May 13;22(10):5162. doi: 10.3390/ijms22105162 (PMC8153024; doi:10.3390/ijms22105162)
Supplement: Supplementary file 1 [file ijms-22-05162-s001.zip › ijms-1211991-supplementary.pdf]

Appendix

Table S1. Metabolite concentration (mean±SE) in roots of potato plants under different P applications

Metabolite (mg g^{-1})	P application in nutrient solution			
	**P0**	**P5**	**P30**	**P40**
**Amino acids**				
Alanine	0.68±0.06	0.47±0.06	0.99±0.51	2.86±0.79
Asparagine	46.58±23.31	3.14±0.38	6.03±1.74	4.61±0.61
Aspartate	6.10±1.80	1.12±0.14	1.83±0.23	1.61±0.13
Glutamate	2.05±0.36	1.56±0.21	1.50±0.41	1.46±0.09
Glutamine	24.23±8.76	2.63±0.24	2.89±0.97	4.15±0.82
Isoleucine	1.01±0.20	0.44±0.03	0.39±0.05	0.73±0.20
Leucine	0.88±0.27	0.25±0.02	0.26±0.01	0.51±0.15
Lysine	0.39±0.00	0.15±0.02	0.15±0.01	0.20±0.05
Phenylalanine	0.54±0.14	0.15±0.01	0.20±0.02	0.27±0.04
Pyroglutamate	45.87±26.53	5.09±1.43	4.19±1.19	5.55±1.31
Serine	1.23±0.04	0.95±0.36	1.29±0.32	1.23±0.41
Threonine	1.03±0.21	0.43±0.07	0.44±0.06	0.43±0.10
Tryptophan	0.16±0.03	0.15±0.01	0.16±0.01	0.18±0.01
Tyrosine	0.53±0.27	0.12±0.01	0.15±0.02	0.20±0.05
Valine	1.28±0.22	0.53±0.02	0.55±0.09	0.93±0.16
γ-amino-butyrate (GABA)	1.85±0.26	1.60±0.12	4.41±1.45	6.95±0.54
**Sugars**				
Fructose	6.87±2.43	25.75±0.56	12.85±3.96	23.93±3.29
Glucose	10.20±3.01	49.85±3.38	17.83±7.06	32.13±7.62
Mannitol	0.59±0.11	1.20±0.14	0.89±0.32	0.93±0.18
Mannose	0.59±0.00	1.79±0.10	0.95±0.31	1.45±0.26
Myo-inositol	0.81±0.40	1.16±0.26	1.03±0.42	1.30±0.41
Sucrose	11.90±1.72	40.85±2.50	34.68±2.98	39.80±2.64
**Organic acids**				
α-Ketoglutarate	0.13±0.07	0.79±0.30	0.53±0.06	
Acetate	1.53±0.12	2.66±0.11	2.04±0.24	2.49±0.16
Formate	0.66±0.02	0.85±0.04	0.71±0.09	0.73±0.03
Fumarate	0.60±0.02	1.00±0.11	0.55±0.05	0.62±0.03
Glycerate	0.16±0.03	0.36±0.02	0.35±0.05	0.42±0.09
Malate	4.33±1.16	34.60±2.06	14.90±3.75	13.23±1.86
Pyruvate	0.04±0.00	0.05±0.00	0.05±0.00	0.05±0.01
Succinate	1.04±0.36	3.38±0.62	1.59±0.23	1.48±0.14
**Other organic compounds**				
1,3-Butanediol	0.06±0.01	0.04±0.01	0.10±0.04	0.11±0.01
Acetaldehyde	0.02±0.01	0.05±0.01	0.01±0.00	0.02±0.00
Acetone	0.02±0.00	0.01±0.00	0.02±0.00	0.02±0.00
Ethanol	0.02±0.01	0.03±0.00	0.02±0.00	0.03±0.00
Glycerine	1.98±0.38	3.55±0.19	2.33±0.31	2.99±0.25
Methanol	3.72±0.09	4.70±0.32	3.11±0.38	3.56±0.29
Choline	3.63±0.69	2.45±0.15	1.84±0.10	2.13±0.16

Table S2. A comparison between oven-dried and freeze-dried leaf samples on total phenolic concentration (mg g^{-1} DM)

Sample	60°C oven-drying	Freeze-drying
1	16.00	16.36
2	10.89	12.79
3	12.17	13.21
4	14.73	14.66
5	11.24	13.78

Table S3. Concentration of nutrients applied in quartz sand for potato seedling germination

**Nutrient**	**Concentration (mg kg^{-1} soil)**	**Nutrient sources**
P	8	$Ca(H_2PO_4)_2$*H_2O
N	300	$Ca(NO_3)_2$* $4H_2O$
K	330	K_2SO_4
Ca	1300	$CaCO_3$, $Ca(H_2PO_4)_2$*H_2O, $Ca(NO_3)_2$*$4H_2O$
S	250	K_2SO_4, $MgSO_4$*$7H_2O$, $CuSO_4$*$5H_2O$, $ZnSO_4$*$7H_2O$, $MnSO_4$*H_2O
Mg	100	$MgSO_4$*$7H_2O$
Cu	0.002	$CuSO_4$*$5H_2O$
EDTA-Fe	0.003	$C_{10}H_{12}FeN_2NaO_8$*$3H_2O$
Zn	0.002	$ZnSO_4$*$7H_2O$
B	0.0006	H_3BO_3
Mo	0.002	Na_2MoO_4*$2H_2O$
Mn	0.006	$MnSO_4$*H_2O

Table S4. Concentration of nutrients applied in the hydroponic system

**Nutrient**	**Concentration (mg L^{-1})**	**Nutrient sources**
P	0 - 40	$Ca(H_2PO_4)_2$*H_2O
N	56.04	NH_4NO_3, $Ca(NO_3)_2$* $4H_2O$
K	104.25	K_2SO_4
Ca	69.05	$Ca(NO_3)_2$* $4H_2O$, $CaCl_2$* $2H_2O$
S	45.01	K_2SO_4, $MgSO_4$* $7H_2O$, $CuSO_4$* $5H_2O$, $ZnSO_4$* $7H_2O$, $MnSO_4$* H_2O
Mg	1.62	$MgSO_4$* $7H_2O$
Cu	0.02	$CuSO_4$* $5H_2O$
EDTA-Fe	5.58	$C_{10}H_{12}FeN_2NaO_8$* $3H_2O$
Zn	0.09	$ZnSO_4$* $7H_2O$
B	0.14	H_3BO_3
Mo	0.13	$H_{24}Mo_7N_6O_{24}$* $4H_2O$
Mn	0.07	$MnSO_4$* H_2O
Na	2.32	$C_{10}H_{12}FeN_2NaO_8$* $3H_2O$
Cl	2.83	$CaCl_2$* $2H_2O$

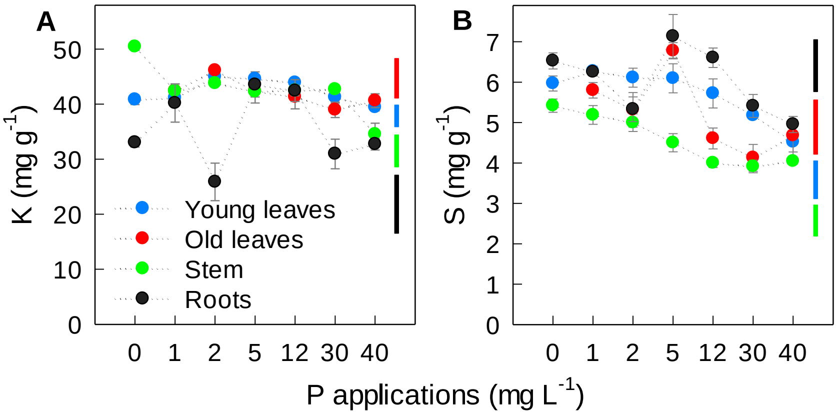

Figure S1. Effect of different P applications on concentrations of K and S in young leaves, old leaves, stem, and roots. For all measured traits, P0 data of old leaves are missing due to insufficient sample material for analyses. Error bars indicate standard error of means (n=4). Vertical bars represent critical value for comparisons among P applications in each plant part by Tukey's HSD test at p≤0.05.

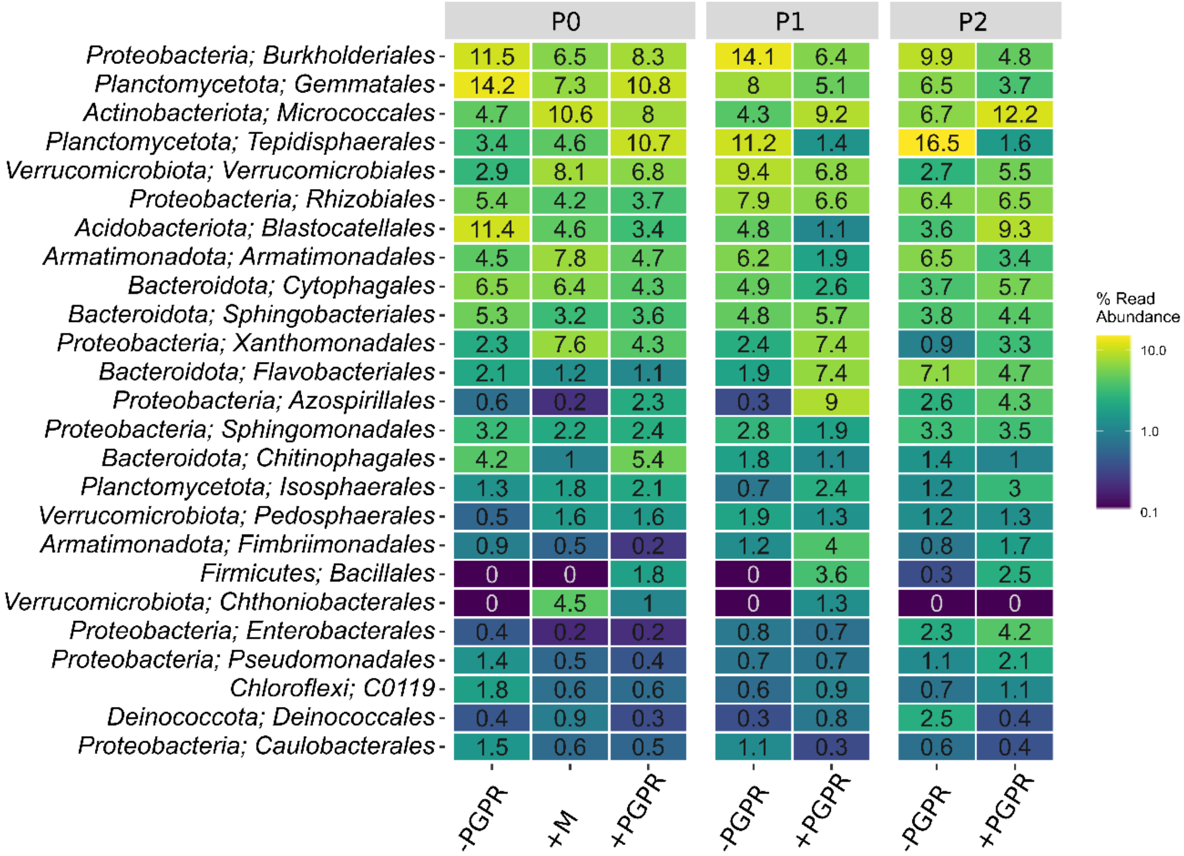

Figure S2. Heatmap depicting the 25 most abundant root-associated bacterial orders occurring in the data set. The shown mean values are based on the calculation of 4 biological replicates (n=4), except for P2+PGPR with n=2 and P1-PGPR with n=3 due to insufficient sample material.

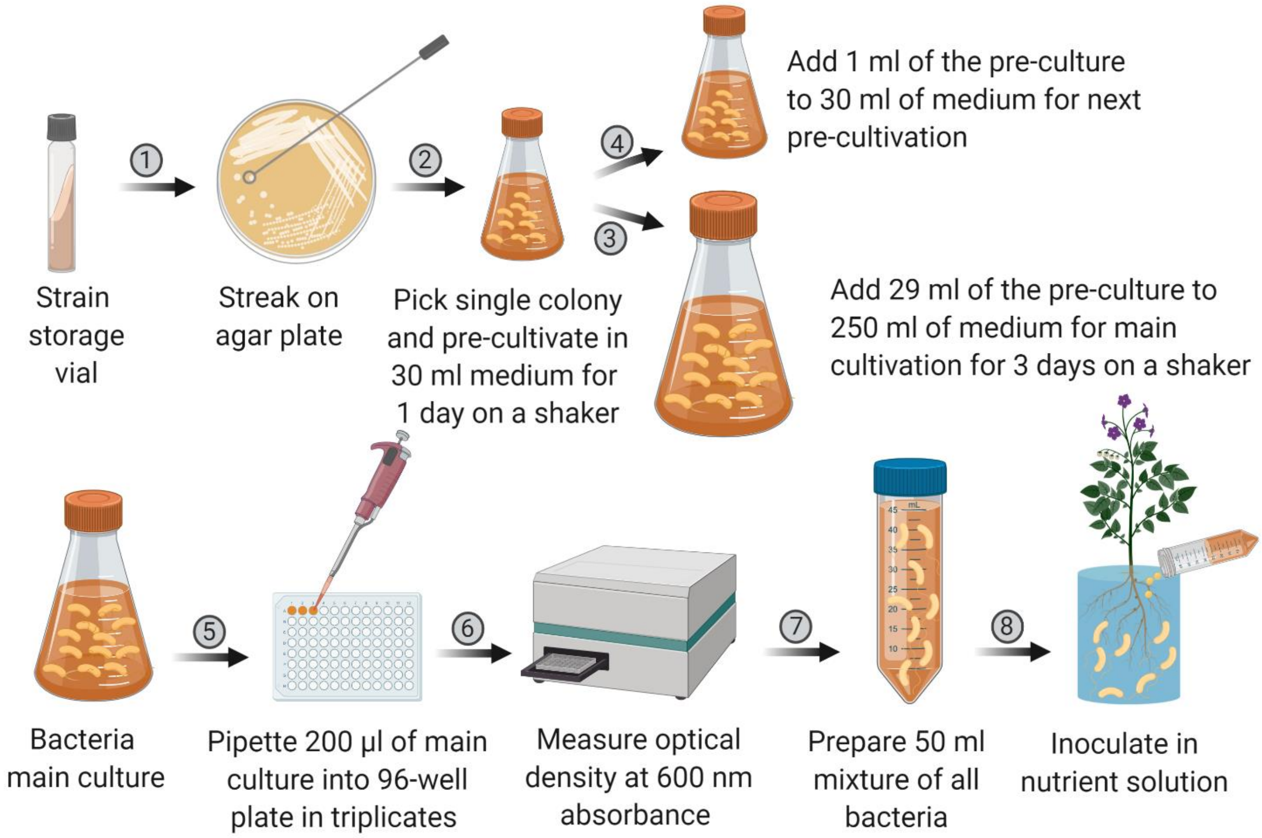

Figure S3. Schematic representation of bacteria cultivation and inoculation procedures. Nutrient broth for each bacteria cultivation was in accordance to supplier's recommendation. Figure was created using BioRender (https://biorender.com, accessed on 14/12/2020) as part of Academic License.
